# Genome-wide translation control analysis of developing human neurons

**DOI:** 10.1186/s13041-022-00940-9

**Published:** 2022-06-15

**Authors:** Érico Moreto Lins, Natássia Cristina Martins Oliveira, Osvaldo Reis, Adriano Ferrasa, Roberto Herai, Alysson R. Muotri, Katlin Brauer Massirer, Mário Henrique Bengtson

**Affiliations:** 1 Department of Biochemistry and Tissue Biology, Institute of Biology, University of Campinas-UNICAMP, Campinas, SP 13083-970 Brazil; 2 Center for Molecular Biology and Genetic Engineering-CBMEG, University of Campinas-UNICAMP, Campinas, SP 13083-875 Brazil; 3 Center of Medicinal Chemistry-CQMED, Structural Genomics Consortium-SGC, University of Campinas-UNICAMP, Campinas, SP 13083-886 Brazil; 4 Graduate Program in Genetics and Molecular Biology (PGBM), UNICAMP, Campinas, SP 13083-886 Brazil; 5 Department of Pediatrics and Cellular and Molecular Medicine, School of Medicine, UC San Diego, La Jolla, CA 92037 Brazil; 6 School of Medicine, Graduate Program in Health Sciences, Pontifícia Universidade Católica do Paraná, Curitiba, PR 80215-901 Brazil; 7 Department of Computer Science, State University of Ponta Grossa-UEPG, Ponta Grossa, PR 84030-900 Brazil

**Keywords:** Translational control, Neural stem cells, Neuron, Neuronal differentiation

## Abstract

During neuronal differentiation, neuroprogenitor cells become polarized, change shape, extend axons, and form complex dendritic trees. While growing, axons are guided by molecular cues to their final destination, where they establish synaptic connections with other neuronal cells. Several layers of regulation are integrated to control neuronal development properly. Although control of mRNA translation plays an essential role in mammalian gene expression, how it contributes temporarily to the modulation of later stages of neuronal differentiation remains poorly understood. Here, we investigated how translation control affects pathways and processes essential for neuronal maturation, using H9-derived human neuro progenitor cells differentiated into neurons as a model. Through Ribosome Profiling (Riboseq) combined with RNA sequencing (RNAseq) analysis, we found that translation control regulates the expression of critical hub genes. Fundamental synaptic vesicle secretion genes belonging to SNARE complex, Rab family members, and vesicle acidification ATPases are strongly translationally regulated in developing neurons. Translational control also participates in neuronal metabolism modulation, particularly affecting genes involved in the TCA cycle and glutamate synthesis/catabolism. Importantly, we found translation regulation of several critical genes with fundamental roles regulating actin and microtubule cytoskeleton pathways, critical to neurite generation, spine formation, axon guidance, and circuit formation. Our results show that translational control dynamically integrates important signals in neurons, regulating several aspects of its development and biology.

**Supplementary Information:**

The online version contains supplementary material available at 10.1186/s13041-022-00940-9.

## Introduction

Several mechanisms have evolved to allow precise and timely control of gene expression, creating multiple layers of regulation such as transcription, splicing, mRNA stability, translation, protein stability, and activation. Moreover, all these layers of regulation need to work in an integrated manner to orchestrate complex biological phenomena. During development, for example, the mere miss-expression of a single gene can have catastrophic consequences for the organism.

Among these processes, recent works have shown that translation control substantially contributes to global gene expression regulation [1]. Although its relative importance, only in the last decade, with the development of Riboseq, it became possible to perform global quantitative measurements of individual transcript translation efficiency and analyze how it is regulated in different physiological conditions [2].

This form of gene expression regulation is particularly important for neuronal cells, which need to manage the dynamic proteome of very long axonal processes, far away from the cellular body. It has been estimated that it would take approximately 12 days to transport protein synthesized at the cell body to distal neuronal sites [3]. Therefore, local protein synthesis can better manage the necessity of fast changes in axonal processes proteome. Besides, each cortical neuron also needs to individually and independently manage, on average, 1000 dendrite connections, which are physically modified with learning and which also store our memories [4]. The mRNAs present at the end of these terminations are tightly regulated by RNA binding proteins and miRNAs to be translated in local ribosomes [5] on-demand upon synaptic activation, contributing to synaptic strength modifications. An essential part of the translation that happens at the synaptic sites, at the end of neurites, is proposed to occur in monosomes due to space constraints [6].

The importance of translation control in neurons seems to extend to the modulation of neuronal differentiation, with several genes that participate in processes that happen during cortex morphogenesis, such as neuronal commitment, differentiation, and migration being translated regulated [7]. Recently, the mechanisms involved in global translation regulation and the full extent of genes and pathways regulated translationally during neuronal differentiation started to be investigated. The works published so far have been using neurons in different stages of in vitro differentiation as a model, since it recapitulates the processes that happen during development. With the current protocols, neuroprogenitors cells (NPCs) take 4 weeks to generate synaptic-competent cortical neurons in vitro [8], with a great number of pathways being dynamic regulated at different differentiation stages.

Grabole 2016 [9] compared, by ribosome profiling, the translatome of TSC2 deficient neuroprogenitors with their normal counterpart and verified a 5′UTR motif-mediated increase in ribosome occupancy in mutant cells. This result indicates that the mTOR pathway is important for the translation regulation of genes during neuronal differentiation. Blair 2017 [10] have shown extensive translation control remodeling during early (15 days) cortical differentiation of neuroprogenitor cells. Part of the observed translation inhibition was proposed to be promoted by alternative 3′UTR extension in neuronal cells. More recently, Rodrigues 2020 [11], using TRAP-sequencing, has compared IPSC-derived NPCs translatome with corresponding 3 weeks differentiated neurons translatome and identified several transcription factors, glycolytic genes, and autism spectrum disorder risk genes as translationally regulated during neuronal differentiation.

In this work, we have investigated genes and pathways subjected to translation regulated during H9 stem cells derived NPC differentiation to latter developmental stage neurons than previously analyzed. Our results offer insights into how translation control contributes to neuronal maturation, neurite extension/guidance, and connections establishment.

## Materials and methods

Detailed experimental procedures are provided in the Additional file 1.

### NPCs generation and differentiation

NPCs were generated as previously described [12], with minor alterations indicated in the Additional file 1. Cells were grown in poly-l-ornithine (10 µg/mL) and laminin (2.5 µg/mL) coated dishes in NBF media. Neuronal differentiation was induced by FGF2 withdrawal when they reached ~ 65% confluency and were maintained in NB media. For NGS analysis, biological replicates NPCs from different passages were differentiated independently and collected on different days.

### Immunofluorescence

Cells were plated onto 13 mm glass coverslips pre-coated with Matrigel (hESC) or poly-l-ornithine/laminin (NPC and Neurons) in a 24-well plate. Cells were fixed in 4% paraformaldehyde for 20 min at room temperature, permeabilized with 0.1% Triton X-100 in 1 × PBS for 15 min, and then blocked with 2% BSA in 1 × PBS for 4 h at room temperature. Primary antibodies in blocking solution were added to the samples and incubated overnight at 4 °C. Secondary antibodies were incubated with samples for 1 h at room temperature. Cells were washed with 1 × PBS and coverslips mounted using ProLong Gold Antifade Mountant with DAPI. Images were acquired using a confocal microscope Leica TCS SP5 II (Leica Microsystems).

### Harvesting and NGS library preparation

NPCs grown in 10 cm dishes were scrapped with polysome lysis buffer (Tris–HCl 20 mM pH 7.5, KCl 1.5 mM, MgCl_2_ 5 mM, 1% Triton X-100, DTT 1 mM, CHX 100 µg/mL) for Riboseq or Trizol for RNAseq. Lysates were digested with RNase I (15U/A260OD) for 30 min at 4 °C as described [13]. Ribosomes were loaded in sucrose cushion and purified through ultracentrifugation. The final ribosome pellet was resuspended in Qiazol. From there on, Riboseq and RNAseq libraries were prepared as described by [14] with minor modifications (see details in Additional file 1: Supplemental Experimental procedures) and sequenced in Illumina HiSeq 2500.

### RNAseq and Riboseq analysis

Raw reads quality was assessed using the software FastQC version 0.11.5 [15]. Cutadapt version 2.4 was used to trim any remaining Illumina adapters demultiplex [16, 17]. In-house scripts were developed to remove artificial poly-C and poly-T bases added by the template-switching technique. The remaining reads were aligned against the contamination reference (rRNA, tRNAs, and mitochondrial coded genes) using the software Bowtie2 version 5.4.0 [18]. To assess the gene expression, it was built a reference list containing all the human transcripts without pseudogenes and 5′ UTR and 3′ UTR of the coding transcripts. The software Kallisto version 0.44.0 was used to build the index [19]. The statistical analysis was conducted with the R package DESeq2 version 3.7 [20]. At least one CDS aligned raw read per gene in all 12 replicates was required to be included in the final background (14,159 genes). The threshold for significant difference was set as twofold-change. Enrichment analysis was done with DAVID [21], Ingenuity Pathways Analysis—IPA (Qiagen), and SynGO [22] databases comparing classified groups list with the background. Contingency tables containing compartment data retrieved from the literature and classified groups p-values were determined using Fisher’s exact test. Heatmaps, graphical plots, and statistical analysis were done using R, GraphPad Prism 6.0, and Microsoft Excel software.

## Results

### Neuroprogenitor cells differentiation into cortical neurons as a model to study translation regulation of genes involved in neuronal processes

**Fig. 1:**
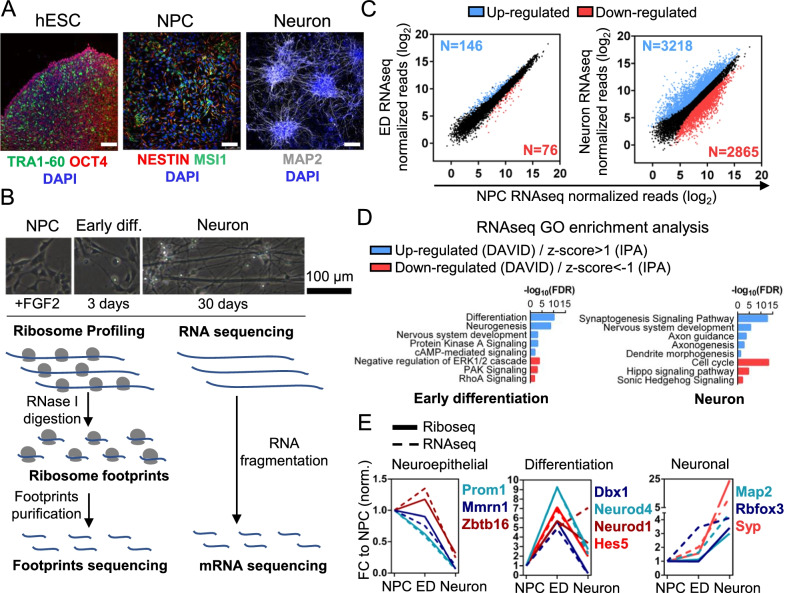
Human neuronal differentiation model and experimental design. **A** H9 human embryonic stem cell line was differentiated into neuroprogenitor cells, which were induced to differentiate into neurons by FGF2 removal for 30 days. Each developmental stage was tested for correspondent markers. hESC (green: TRA1-60; Red: OCT4), NPC (Red: NESTIN) and Neuron (white: MAP2). Blue: DAPI. Scale bars = 100 µm. **B** Experimental design. Samples from NPCs in different points of differentiation were collected for Ribosome Profiling and RNA sequencing analysis. **C** Transcriptomic analysis of differentiating NPCs. RNAseq libraries prepared in duplicates from ED and Neurons were compared with NPC libraries. Differentially expressed genes are indicated according to FC > 2, FDR < 0.01. Blue: upregulated; Red: downregulated. **D** DAVID and IPA Gene Ontology analysis of RNAseq differentially expressed transcripts in ED and Neurons (FDR < 0.01). **E** Differentiation markers analysis. Plot with Riboseq and RNAseq fold-change values (normalized to NPC) of neuroepithelial, neural differentiation, and canonical neuronal markers

Neuroprogenitor cells (NPC) were differentiated from H9 human embryonic stem cells using a SMAD dual inhibition strategy [8] (Additional file 2: Fig. S1A). The generated NPCs were positive for the progenitor marker Nestin and upon differentiation induction by FGF removal, developed neural projections, and expression of synaptic neuronal markers (Figs. 1A, 3A; Additional file 2: Fig. S1B).

For translation efficiency analysis we selected 3 conditions: NPC, 3 days (Early differentiation—ED), and 30 days (neuron) after differentiation induction (Fig. 1B). We simultaneously collect material to perform transcriptome (RNAseq) and translatome (Ribosome Profiling—Riboseq) analysis to be able to measure translation efficiency (TE = Riboseq reads/RNAseq reads) of each expressed gene. The quality parameters for the Riboseq and RNAseq libraries confirmed a high correlation between biologic replicates (Additional file 4: Fig. S3).

We first performed a transcriptome analysis to validate our model (Fig. 1C). During the first 3 days of differentiation, only a few genes (222) had their expression significative modified, in contrast to 30 days of differentiation (around 6000 genes). As expected, differentiation markers analysis (Fig. 1E; Additional file 3: Fig. S2) indicated a gradual reduction of neuroepithelial markers, greater induction of early differentiation markers in 3 days, and high induction of neuronal, synaptic, and glutamatergic makers after 30 days of differentiation. The high expression of doublecortin suggests that even after 30 days of differentiation, the generated neurons are still going through the maturation process. Comparison between transcriptome data from previously published works with ours, and among themselves [10, 11] (Additional file 5: Fig. S4), pointed out little correlation between regulated genes in the different time points, indicating that the transcriptome is dynamic regulated during differentiation. Therefore, the chosen 30 days differentiation time point could reveal new translation-regulated genes not encountered in the previously published work.

Gene Ontology analysis of differentially expressed genes (Fig. 1D) shows a significant induction of genes functionally related to nervous system development, dendrite morphogenesis, axonogenesis, axon guidance, and synaptogenesis. Together with the cellular morphology changes observed in cultured cells (Additional file 2: Fig. S1B), these results indicate that translation efficiency analysis in the selected time point could help elucidate how translation control contributes to the regulation of the above neuronal processes.

### Translation control significantly contributes to differential gene expression between NPC and neurons

**Fig. 2:**
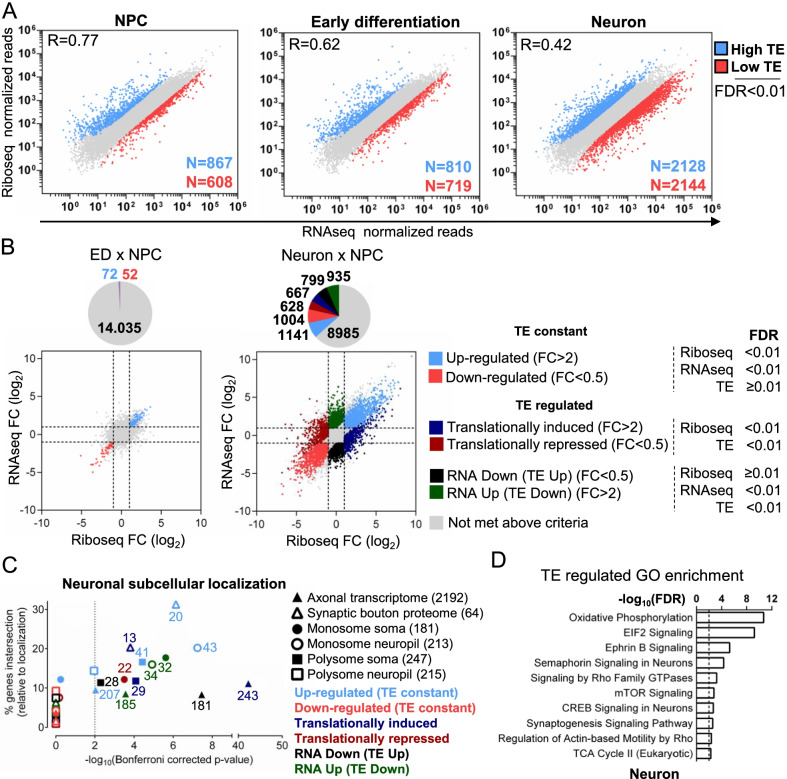
Translational Control analysis of genes expressed during human neuronal differentiation. **A** Ribosome Profiling versus RNA sequencing expression plots at selected differentiation stages. Blue: High translation efficiency genes (TE > 3); Red: low translation efficiency genes (TE < 0.33), FDR < 0.01. **B** RNAseq versus Riboseq fold-change plots comparing ED and Neurons with NPC. Genes were classified according to their regulation with the parameters indicated criteria. Blue represents Up-regulated genes, with no change in translation efficiency; Red indicates Down-regulated genes, with no change in translation efficiency; Dark blue indicates genes with increased translation efficiency and significative increase of total riboseq counts between cells; Dark red indicates genes with decreased translation efficiency and significative decrease of total riboseq counts between cells; Black indicates genes with increased translation efficiency without a significative increase in total riboseq counts between cells; Green indicates genes with decreased translation efficiency without significative decrease of total riboseq counts between cells. Gray indicates genes without regulation. **C**) Subcellular localization analysis of translation-regulated transcripts. Genes from each regulatory category in **B** were compared with human axonal transcriptome [25], synaptic bouton proteome [26] and monosome/polysome enriched transcripts in soma/neuronal neuropil [79]. Data is presented as Fisher exact test p-values (X-axis) to the percentual intersected relative to total genes in the subcellular localization category list (Y-axis). **D** IPA GO enrichment analysis of TE regulated genes in Neurons (TE FC > 2, FDR < 0.01)

Next, we compared mRNA and translation levels of 14,159 genes for which we obtained a significant number of counts, after sequencing a minimum of 20 million unique mapped reads per library (Additional file 8: Table S1). In NPC, RNAseq and Riboseq reads showed a high Pearson correlation of 0.77 (R = 0.77), which is similar to the previously reported value for NPC [10] and mammalian cells in general [23]. However, this correlation progressively and drastically modified from NPC, Early Differentiation (ED) to neurons (R = 0.42), indicating that neurons greatly rely on translation control to regulate their protein expression (Fig. 2A).

Since none of the regulated genes in ED samples showed modifications in translation efficiency (TE), we will discuss the neuron to NPC comparison, where thousands of genes changed their TE (Fig. 2B). A summary of the different translation regulation classification and criteria used in our study is provided in Fig. 2B. For 2145 differentially expressed genes between these two cells, there is a perfect direct correlation between their RNAseq and Riboseq regulation data (genes up or down-regulated, TE constant category). For 3029 genes, however, there is a statistically significant TE modification between the 2 conditions. Surprisingly, for more than half of these genes (1734 genes), Riboseq counts did not change significantly meanwhile mRNA abundance either increased (RNA up, TE down category) or decreased (RNA down, TE up category). The elevated number of genes found in this category, known as “translation buffering” [24] could be due to the presence of mRNA stored translationally inhibited in neuronal granules in neurons. For 1295 genes, their TE significantly changed in neurons due to translation up or down-regulation in relationship to corresponding mRNA levels (genes translationally induced and translationally repressed categories, respectively).

To gain more insight into the subcellular distribution of translation-regulated transcripts, we compared our data with the following publicly available datasets: axonal transcriptome [25], synaptic bouton proteome [26], neuronal soma, and projections Riboseq (neuronal neuropil) [27], (Fig. 2C; Additional file 10: Table S3). After bioinformatics comparative analysis of these datasets, we found a statistically significant enrichment of translationally regulated genes whose transcripts are found in axons in almost all categories. Around 11% of the 2192 genes present in axons are translationally induced, corresponding to 36% of all translationally induced genes (Fig. 2B). More than half of the proteins detected in the synaptic bouton proteome are either upregulated (TE constant) between NPC and neuron or are translationally induced. Interestingly, there is an enrichment of translationally inhibited transcripts in monosomes in general (translationally repressed and RNA up, TE down); meanwhile, polysomes are enriched in transcripts upregulated or translationally upregulated (Translationally induced and RNA down, TE up).

Next, we performed a GO enrichment analysis (using Ingenuity Pathways Analysis system—IPA) on the genes that changed translation efficiency between cells to investigate if any neuronal pathway or process is particularly regulated translationally (Fig. 2D; Additional file 9: Table S2). Interestingly, the processes of oxidative phosphorylation, synaptogenesis, and pathways that modulate actin polymerization for neuritogenesis and axon guidance are highly enriched in translationally regulated genes. In addition, pathways that participate in translation control such as mTOR and EIF2 signaling are also translationally regulated and may be in part responsible for the intense translation program modification observed between compared cells. Translationally controlled genes on these pathways and processes are discussed in the next sections.

Therefore, translation regulation significantly contributes to differential gene expression between NPC and 30 days Neurons and seems to participate in regulating important neuronal pathways and processes involved in neuronal development.

### Translation control regulates essential synaptic genes necessary for synaptic transmission

**Fig. 3:**
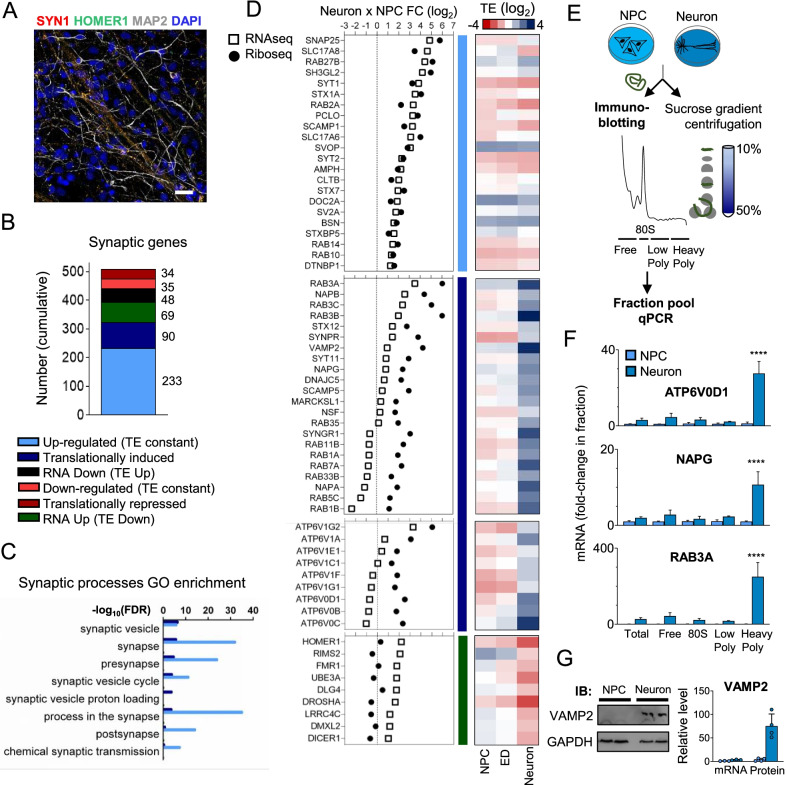
Translational Control of synaptic genes. **A** Immunofluorescence showing positive presynaptic and post-synaptic markers in 30 days Neurons (green: HOMER1; Red: SYN1; white: MAP2. Blue: DAPI. Scale bars = 100 µm. **B** Classification of the differential expressed synaptic genes between NPC and neuron accord with their mode of regulation. **C** SynGO enrichment analysis and total Unique terms quantification of the translation-regulated genes in Neurons in relationship to NPCs. Categories with significant enrichment (FDR < 0.01) in SynGO processes are shown. Dark blue bars: Translational induced category; Light blue bars: up-regulated (TE constant) category. **D** RNAseq and Riboseq fold-change plots of synaptic vesicles genes Up-regulated and translationally regulated in Neurons compared to NPC. Corresponding TE heatmap of v-ATPase complex subunits, selected RNA Up, TE Down genes, and selected translationally induced genes at each differentiation time-point are indicated. Data are presented as log_2_ of TE value at each time point. **E** Experimental design used to confirm translation regulation of selected genes. **F** ATP6V0D1, NAPG, and RAB3A mRNA are strongly associated with heavy polysomes in neurons. The graphic represents an average of 3 independent experiments. **** indicates p < 0.0001. **G** VAMP2 is more induced at the protein level than at mRNA level during the differentiation of NPCs into neurons. Comparative protein levels were determined by western blot meanwhile mRNA levels were determined by qPCR. The analysis was performed in 4 independent biological samples

In the selected time point, synaptic markers are detected in a pattern that indicates that synaptic connections are forming (Fig. 3A). To better understand which synaptic genes and processes are translationally modulated, we reanalyzed our data using SynGO, a GO database focused on synaptic processes [22]. Most of the genes involved in synapse transmission are strongly induced during differentiation but not translation regulated (Fig. 3B). However, almost half of the identified differentially expressed synaptic genes are translationally regulated. For 90 of these genes, translation efficiency is increased with a significant increase in Riboseq reads (translationally induced category), meanwhile, for 34 genes, translation is down-regulated (translationally repressed category). For 117 genes, there is either mRNA up or down-regulation, without a significant change in riboseq reads (RNA up, TE down; RNA down, TE up categories).

Synaptic processes are significantly enriched in induced genes without translation regulation (upregulated TE constant) and genes translationally induced (Fig. 3C). For the GOs “synaptic vesicle” and “synaptic vesicle proton loading”, translationally induced genes are even more enriched than the set of upregulated genes with constant TE. Some translationally induced genes on these GOs code for proteins related to the SNARE complex (*N*-ethylmaleimide-sensitive factor attachment protein receptors) (Fig. 3D), which mediate vesicle trafficking and membrane fusion. In neurons, the SNARE complex is essential for calcium-triggered synaptic vesicles exocytosis and works together with the small GTPase RAB family members (Rab3a/b/c and Rab27b) [28, 29]. This complex is formed by 3 families of proteins: synaptobrevin, syntaxin, and synaptotagmin. In our data, synaptobrevin 2 (Vamp2), syntaxin 12 (stx12), synaptotagmin 11 (Syt11), and all members of SNARE disassembly/recycling complex formed by NSF, NAPA (alpha-snap), NAPB (beta-snap), and NAPG (gamma-snap) are translationally induced (Fig. 3D). Among these genes, Vamp2 is essential for synaptic transmission [30] and it is very strongly translation induced (TE = 1.5 in NPCs and TE = 14 in neurons). Other synaptic SNARE genes that work together with Vamp2, such as Syt1A and SNAP25, are strongly upregulated without translation regulation.

From the RABs involved in synaptic transmission, all 3 Rab3s (A, B, and C) are strongly translationally induced meanwhile Rab27a/b are very low expressed and do not seem to be translation-regulated. In addition, several other Rab family members are highly translationally regulated, constituting one of the most translation-regulated gene families in our dataset: RABs 1A/B, RAB5c, Rab7A, RAB11B, RAB33B, and RAB35 (Fig. 3D). The regulated RABs are involved in several aspects of neuronal biology: synaptic vesicle recycling (RAB5C, RAB33B, and RAB35), retrograde neurite transport (RAB5c and RAB7), and neurite outgrowth (RAB7, RAB11, and RAB35) [29].

Almost all regulated genes in “Synapse vesicle proton loading” GO are translationally induced. This GO comprises vacuolar ATPases that promote vesicle acidification necessary for neurotransmitter loading into synaptic vesicles [31] (Fig. 3D).

Next, to confirm some of these results, we randomly selected translation-induced genes for further validation experiments. In these experiments, we used two different methods on a non-redundant list of genes, to maximize the number of interesting candidates tested. Confirmation in any of these methods provides further evidence of the translation regulation.

The mRNA distribution of ATP6VOD1, NAPG, and RAB3A was analyzed in sucrose gradient fractions obtained from 3 new NPCs and neuronal samples (Fig. 3E). As expected for differentially translated regulated genes, they were enriched in the high polysomal fraction in neurons but not in NPCs, suggesting that they have a higher translation efficiency in neurons (Fig. 3F). For VAMP2, we have compared protein and mRNA levels in NPCs and neurons (Fig. 3G; Additional file 7: Figure S6). Although mRNA was induced only around 3 folds during differentiation, protein level increased more than 70X, corroborating with the results of the translation regulation analysis.

Altogether, our data indicate that translation control is specially used to modulate synaptic gene expression, regulating essential proteins and processes necessary for synaptic transmission.

### Translation control contributes to the regulation of neuronal cell metabolism

**Fig. 4:**
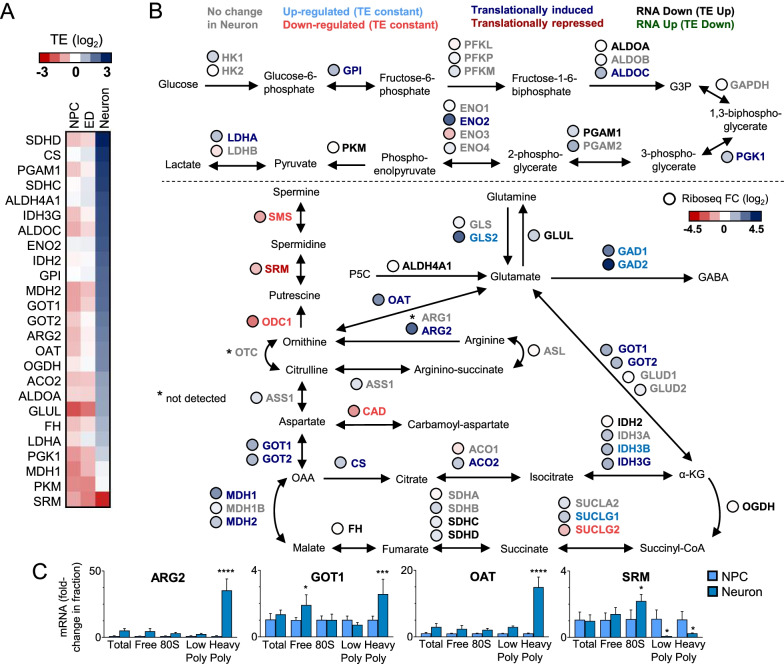
Translational Control of neuronal metabolic genes involved in Glycolysis, TCA, Urea cycle, and glutamate metabolism. **A** TE Heatmap showing significantly regulated genes (TE FC > 2, FDR < 0.01) involved in glycolysis, TCA cycle, and glutamate metabolism in Neurons. **B** Glycolysis, TCA, Urea cycle, and glutamate/GABA metabolic map. Gray: No regulation in neurons; Blue: Up-regulated genes; Red: Down-regulated genes; Dark blue: Translationally induced genes; Dark Red: Translationally repressed genes; Black: RNA Down, TE Up genes; Green: RNA up, TE down genes. Circles: Riboseq FC Heatmap for each gene. **C** ARG2, GOT1, and OAT and SRM mRNA are more associated with heavy polysomes in neurons; meanwhile, SRM is more associated with heavy polysomes in NPCs. The graphic represents an average of 3 independent experiments. Asterisks indicates: *p < 0.05, **p < 0.01, ***p < 0.001, ****p < 0.0001

The obtained RNAseq data confirm downregulation of glycolysis genes, with the absence of up-regulation of tricarboxylic acid (TCA) genes, previously reported to occur during the metabolic shift (glycolysis—NPC to OxPhos—Neurons) that follows neural differentiation [32]. However, our Riboseq data indicates that several of these genes have their translation efficiency and total translation increased upon differentiation (Fig. 4A, B). The glycolysis and TCA genes under translational regulation in our dataset are different from those reported by Rodrigues et al. [11] (Additional file 11: Table S4), which probably is due to the different stages of neuron differentiation analyzed. Both results suggest that translation control is an important layer of regulation that contributes to adjusting the OxPhos pathway in neurons during development. Besides glucose catabolism, other metabolic enzymes are either translationally induced or repressed in neurons in our dataset, particularly genes involved in the ornithine cycle (Urea cycle) and aspartate metabolism.

In the Urea cycle, there is a strong translation induction of ARG2 and OAT (translationally induced category) enzymes with a simultaneous translation down-regulation of SRM (translationally repressed category) and transcriptional downregulation of ODC1 and SMS enzymes (downregulated TE constant category). These regulations suggest that in neurons there is an increase in ornithine production from arginine, with inhibition of its usage in polyamines synthesis (putrescine, spermidine, and spermine) and prioritization of its use to produce glutamate-semialdehyde in a reaction that may convert alpha-ketoglutarate into glutamate. Glutamate-semialdehyde can also be converted into glutamate by the ALDH4A1 enzyme.

Translational up-regulation of GOT1/2 enzymes (translationally induced category) with transcriptional downregulation of CAD (downregulated TE constant category) suggests that aspartate usage to generate oxaloacetate is increased in neurons, in a reaction that simultaneous convert alpha-ketoglutarate into glutamate. Although alpha-ketoglutarate from TCA can be used as a glutamate source to glutamatergic neurons, it is believed that most of the glutamate used as a neurotransmitter by these neurons come from glutamine produced by astrocytes (Glutamine-Glutamate cycle) [33]. This happens due to the absence of pyruvate carboxylase enzyme expression in neurons and consequently the inability to synthesize oxaloacetate to maintain the TCA cycle depleted of alpha-ketoglutarate [34]. In neuronal cultures derived from NPC, astrocytes differentiate and accumulate much later. Therefore, we speculate that in the absence of sufficient glutamine provided by astrocytes, neuronal cells may increase metabolic usage of amino acids as a source of glutamate.

To validate the translation regulation of ARG2, GOT1, OAT, and SRM, we analyzed their mRNA distribution in a sucrose gradient. The results confirmed that ARG2, GOT1, and OAT mRNA are more associated with high polysomal fractions in neurons than in NPCs. Furthermore, as expected for a translation inhibited transcript, SRM mRNA was detected mostly in the free and 80S fractions in neurons (Fig. 4C).

Overall, our results show that neurons rely on the translation regulation of metabolic enzymes to fine adjust their metabolism according to cell demand.

### Translation control participates in the regulation of actin and microtubule cytoskeleton pathways critical for neuronal projections

The neuronal cytoskeleton is actively modeled to allow dendritogenesis, axon growth, and guidance during development. Actin in the growth cone, present on the tip of axons or dendrites, can be polymerized or depolymerized in response to extracellular cues to guide neurites' extension to their right destination. In mature neurons, cytoskeleton structure changes in response to synapse activity and is implicated in new synapses formation.

**Fig. 5:**
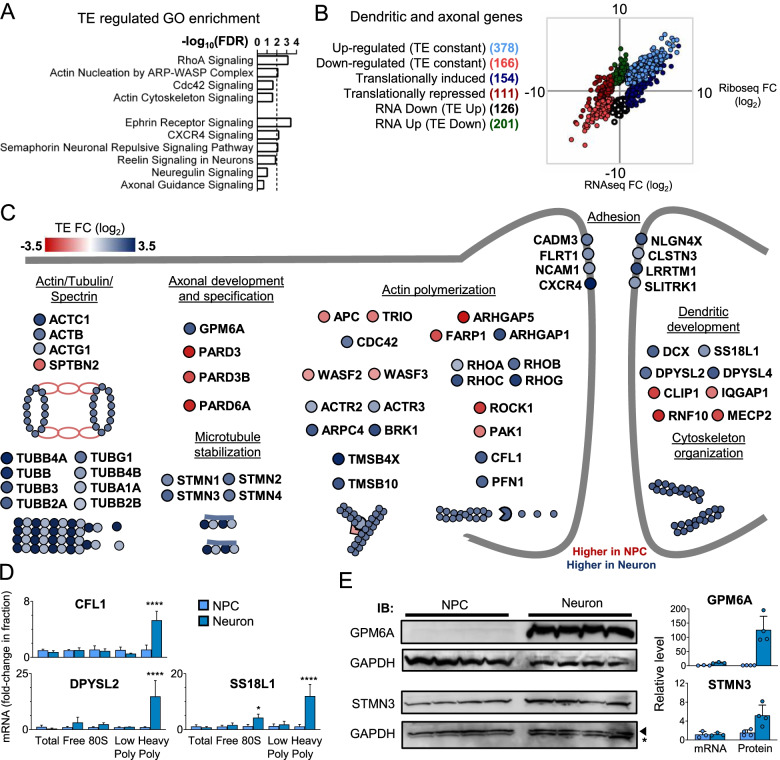
Translational Control of neuritogenesis, axon guidance, and cytoskeleton genes in Neurons. **A** IPA GO analysis of Actin cytoskeleton and neurite guidance processes enriched in translation-regulated genes. The dashed line marks FDR < 0.01. **B** RNAseq versus Riboseq FC plot of dendritic and axonal AMIGO GO processes genes regulated between neurons and NPCs. Different translation regulation categories are indicated in the figure. **C** TE heatmap of cytoskeleton regulators and structural genes. Data are presented as the TE fold-change value in Neurons compared to NPC. **D** CFL1, DPYSL2, and SS18L1 mRNA are strongly associated with heavy polysomes in neurons. The graphic represents an average of 3 independent experiments. Asterisks indicate **** p < 0.0001. **E** GPM6A and STMN3 are more induced at the protein level than at the mRNA level during the differentiation of NPCs into neurons. Comparative protein levels were determined by western blot, meanwhile mRNA levels were determined by qPCR. The analysis was performed in 4 independent biological samples

Interestingly, IPA GO enrichment analysis on translation-regulated genes shows strong enrichment of GOs associated with actin cytoskeleton regulatory pathways and upstream axon/dendrite development and guidance pathways (Neuregulin, Reelin, Semaphorin, CXCR4, and Ephrin signaling) (Figs. 2D, 5A, B).

To expand these findings, we performed a careful search of all genes belonging to the AMIGO [35] GO terms axogenesis, dendritogenesis, and axon guidance and investigated their translation regulation. This analysis revealed that an important fraction of these genes is translationally regulated representing more than half (592 genes) out of 1136 differentially expressed genes in these processes. Furthermore, around 25% (265 genes) are in the translationally induced or repressed category (Fig. 5B).

Many of the translated regulated genes are key hubs on the investigated processes, suggesting that translation control is specially used by neurons to modulate cytoskeleton structural changes necessary to neuritogenesis and neurite guidance (Fig. 5C).

Two of the 3 small GTPases from the Rho family that play crucial roles in mediating actin cytoskeleton remodeling are translation modulated: CDC42 (translationally induced) and RhoA (RNA down, TE up). In neurons, RhoA activation is associated with growth cone collapse, meanwhile, Cdc42 is downstream of attractive clues [36]. RhoA activates ROCK, which phosphorylates LIMK, promoting CFL phosphorylation inhibiting its actin depolymerization activity. LIMK can also be phosphorylated by Pak1, which is downstream of Rac1 GTPase (revied in [37]). Our data show that both ROCK1 and Pak1 are translationally repressed while CFL is translationally induced. CFL has an essential role in regulating the actin cytoskeleton during growth cone elongation [37]. Besides this, 2 GTPases close related to RhoA, and also involved in actin cytoskeleton remodeling, RhoB and RhoC, are equally translationally induced. Several Rho family modulating GEFs and GAPs are translation regulated, such as TRIO, ARHGAP1, ARHGAP5, and FARP1. FARP1, a RAC1 GEF, is implicated in the assembly and disassembly of dendritic filopodia, formation of dendritic spines, and regulation of dendrite length [38, 39].

Some transcripts from proteins directly involved in actin polymerization are also regulated. Profilin binds monomeric actin and catalyzes the exchange of ADP for ATP, promoting actin polymerization into actin barbed ends. This reaction is stimulated by ARP 2/3, WASP, and WAVE complexes. Arp2/3 complex is one of the most important regulators of dendrite spine growth [40]. Profilin, BRK1 (part of WAVE complex), ARPC4, ACTR2, ACTR3 (part of ARP complex) have increased TE; meanwhile, the Wasp complex members WASF2 and WASF3 have a slight decrease in TE. Proteins from the thymosin family sequester monomeric actin, inhibiting actin polymerization [41]. In our data, the thymosin family members TMSB4X and TMSB10 have increased TE in neurons (translationally induced category).

Besides actin cytoskeleton remodeling, proper axon and dendritic arbor morphology rely on careful microtubule polymerization, stabilization, severing, and depolymerization. Likewise, we found the translation regulation of several key proteins that participate in these processes (Fig. 5C).

CRMP2 (DPYSL2) and CRMP3 (DPYSL4), both translationally induced in neurons, work on microtubule cytoskeleton polymerization [42] and are essential for semaphorin class 3 signaling and downstream remodeling of actin and microtubules [43]. In addition, they are major modulators of dendrite development, neuronal growth cone collapse, and axon guidance [44].

Doublecortin is a microtubule-binding protein localized at the ends of neuritic and leading processes, where it regulates microtubule organization and stability, regulating neuron migration and dendrite growth during development[45, 46]. In our data, it is translationally induced in developing neurons.

IQGAP1 is a key regulator of dendritic spine number, morphology, and extension. In the microtubule cytoskeleton, it works cooperatively with its interaction partner Clip170 (Clip1) and APC to regulate microtubule dynamics and stabilization [47]. All three genes have a decreased TE in neurons (IQGAP1 and Clip170 translationally repressed; APC RNA up, TE down). In the actin cytoskeleton, IQGAP1 works in complex with Cdc42 and Rac1 to stimulate actin assembly by N-WASP and the Arp2/3 complexes [48].

As shown in Fig. 5C, transcripts from the Stathmin family (STMN3, STMN2, STMN1) increase their TE (translationally induced category) in developing neurons. Proteins from this family constitute a hub that relays and integrates various signaling pathways and is involved in microtubule dynamics by promoting depolymerization of microtubules or by preventing polymerization of tubulin heterodimers [49]. Interestingly the neuronal-specific STMN3 (also known as SCLIP) [50] is regulated exclusively translationally between NPC and Neuron in our dataset. This gene has been implicated in controlling growth cone expansion, axon morphogenesis, specification, branching [51], and dendritic maturation [52].

Members of the Par polarity complex—PARD3, PARD3B, and PARD6A—are translationally repressed in developing neurons. This complex is essential to establish neuronal polarity, axon specification, and dendritic spine formation [53, 54]. Pard3 is localized in the axon, especially at the growth cone, but is excluded from neurites that will become dendrites [55]. PARD3 local translation is also required for NGF-induced axon outgrowth [56].

Some genes with a less clear molecular mechanism of action but fundamental in controlling neuritogenesis were also found to be translation regulated. Some examples of translation-induced genes in developing neurons in relationship to NPCs are GPM6A, SS18L1 (also known as CREST), and RNF10. GPM6A is required for normal axonal extension and guidance [57], filopodia outgrowth, motility [58], spines, and synapse formation [59]. The transcriptional activator SS18L1 (CREST) is part of the neural progenitor Brg/Brm-associated factor (npBAF) complex and is required for calcium-dependent dendritic outgrowth and branching of cortical neurons [60]. RNF10 is a ring finger protein involved in synapse-to-nucleus signaling downstream to NMDA receptors. It regulates spine density, neuronal branching, and dendritic architecture in hippocampal neurons [61].

To validate some of our findings, we randomly selected 5 translated regulated genes (CFL1, DPYSL2, SS18L1, STMN3, and GPM6A). The first 3 were tested by qPCR analysis of sucrose gradient fractionations of ribosomal complexes (Fig. 5D). They all showed a greater mRNA association with heavy polysomal fraction in neurons when compared to NPCs. The last 2 genes were tested by comparing protein induction with mRNA induction in neurons and NPCs (Fig. 5E). Both seem to be greater induced in protein levels than in mRNA levels in neurons, suggesting that they are translated regulated.

The IPA GO analysis also identified translation modulation enrichment in pathways known to control neuronal circuit formation through cytoskeleton reorganization, such as mTOR, Wnt, NGF, and CREB (Additional file 6: Fig. S5), indicating that translation control mechanisms may participate in tuning these pathways for proper neuron development.

Taken together, our data suggest that translation control mechanisms play an important role in regulating actin and microtubule cytoskeleton pathways that are critical to neurite generation, spine formation, polarization, axon guidance, and circuit formation (Additional files 7, 8, 9, 10).

## Discussion

Translational control of individual transcripts has been previously implicated in neuronal differentiation, axon guidance, synapse regulation, and memory formation. Only recently it has been possible to search for a comprehensive global picture of all genes and pathways regulated by translation during neurogenesis. Comparison between published earlier NPC to neuron differentiation transcriptomes [10, 11] (Additional file 5: Fig. S4) and translatomes show small overlap between them, indicating that both transcription and translation are dynamically regulated during neurogenesis. Therefore, an effort to analyze multiple time points of differentiation, the same way as done for transcription [62], will be necessary to fully understand the temporal participation of translation regulation in neurogenesis.

In this work, we have specifically selected later differentiating cortical neurons, in the stage of neurite extension, migration, and connection establishment to investigate the role of translation control on those processes. The results obtained in our work indicate that developing neurons especially rely on translation control to fine adjust metabolism, regulate genes fundamental for synaptic transmission and modulate important hubs in actin and microtubule cytoskeleton pathways which are critical for neurite generation, spine formation, polarization, axon guidance, and circuit formation. Most of the translation-regulated genes found in our work were not observed to be differentially regulated on those published work, on the same differentiation model but in different points of differentiation (Additional file 11: Table S4).

Previous reports have shown specific translation regulation of neural actin cytoskeleton in developing and adult neurons. Slit2 was shown to relieve Cofilin translation inhibition caused by the microRNA mir182 during axon guidance [63]. β-actin mRNA translation is controlled by different RBPs: ZBP1 during growth cone turning [64], HuD [65], hnRNP R [66], i.e.) and is induced by netrin-1 signaling [67]. RhoA translation is induced by Sema3A, and its regulation is required for growth cone collapse [68]. Albeit in minor numbers, individual genes in the microtubule cytoskeleton are also reported to be translationally controlled. For instance, the microRNA mir29a modulates axon branching by regulating doublecortin translation in primary neuron culture [69]. Our results confirm the translation regulation of all the above genes in our model and expand the list of targets, indicating an intense, widespread translation modulation of cytoskeleton genes during neurogenesis and circuit formation. More recently, Cagnetta et al. [70], have used proteomics to analyze nascent protein synthesis in retinal growth axons of Xenopus subjected to different cue stimuli (Netrin, Semaphorin 3A, and BDNF). In this study, a few cytoskeleton genes (Tuba4b, Tubb4a, ACT2, CDC42, and CFL-1, e.g.) were found to be fast induced/repressed upon stimulus, with a small overlap with our data. Although the cellular models are very different (Xenopus retinal Axon X human entire cortical neuron), it is interesting to verify that translation regulation of these genes is conserved and may participate in axon guidance.

In addition, our results suggest a role for translation regulation of vesicle genes in synapse maturation, particularly members of the snare complex, small GTPase Rab family, and ATPases involved in acidification and neurotransmitter loading. Recently, it was shown that Rbfox1 RBP regulates synaptic transmission through modulation of Vamp1 mRNA translation/stability [71], indicating that translation regulation of snare proteins may be a common mechanism used by neurons to modulate synapse transmission.

Translation control of the identified pathways may help promote fast adjustments in response to stimuli, such as metabolic changes, synapses depolarization, or axon guidance. Genes from housekeeping pathways and processes from the neuronal soma are less likely regulated translationally. Further work is necessary to define how translation control of the identified genes occurs, which RBPs/miRNA are involved, what regulatory elements are present on these transcripts, and the role of translation control pathways such as mTOR, EIF2, and EIF3 in the process. Both mTOR [72] and EIF2 [73] are known to participate in regulating neurite projections and wiring in neurons and probably are regulating some of the genes identified by our study. Our data also showed intense TE modification of EIF3 subunits. Interestingly, this translation initiation factor binds to 5′UTR mRNA structures and induces cap-independent translation initiation or promotes translation inhibition of target mRNAs [74]. It may promote bypass of 5′UTR uORFs translation inhibition. Recent work indicates that 5′UTR uORFs and alternative 3′UTR may contribute to differences in translation efficiency of genes between developmental stages [10, 75] by adding or removing miRNA and RBP binding sites. Differences in the presence of these regulatory elements between genes from the same family may help to explain why some of them are regulated meanwhile others are not. It will be interesting to investigate how alternative structural regulatory elements participate in the translation efficiency differences observed.

One limitation of our study is that it was performed in cell cultures where different processes are happening simultaneously, making it hard to identify the upstream signals responsible for regulation. Nevertheless, our work provides a list of genes and processes for further investigation and search for novel biological mechanisms.

Another question for future research is how the translation efficiency of some genes is regulated by cells to compensate for the decrease/increase of transcription (“translation buffering”) or to compensate for modifications in protein turnover during differentiation. In the latter case, a comprehensive proteomics analysis of the translationally regulated genes products will be necessary to quantify the frequency of this form of regulation.

Although the regulations reported here occur during development, they may also participate in adult neuronal processes. Some of the regulated genes are involved in cognition (IQGAP1 [76], e.g.), synaptic plasticity (RhoB [77], e.g.), and memory (SS18L1 [78], e.g.). Intriguing, several of these genes are known to be mutated in neuronal diseases, which brings into question what the role of translation regulation on these diseases is, and if it can be manipulated to benefit patients.

In summary, our work provides evidence that neurons extensively rely on translation regulation to modulate genes and pathways necessary for their development and function as metabolism, synapses, and cytoskeleton regulated processes.

## Supplementary Information

**Additional file 1:** Supplemental experimental procedures.

**Additional file 2: Fig. S1**. hESC differentiation into NPCs, and neuronal differentiation. A) NPC generation steps, as detailed in methods. B) Light microscopy images showing the timecourse phenotype of neural cells after NPC differentiation induction by FGF removal.

**Additional file 3: Fig. S2**. RNA sequencing expression heatmap and clustering classification of canonical progenitor, neuroepithelial, differentiation, neuronal, synaptic, and glutamatergic markers.

**Additional file 4: Fig. S3**. Quality control of the NGS libraries. A) Optimization of RNase I digestion protocol for ribosome footprint generation with Hek293T samples. B) PCA of NGS biological replicates produced in this study. C) Flowchart of filters applied for Translational Efficiency classification. D) IGV aligned STAR reads tracks comparing Riboseq and RNAseq libraries.

**Additional file 5: Fig. S4**. Comparison of transcriptionally regulated genes between our dataset and previously published data, obtained with cells in different days of NPC differentiation into neurons. A) Venn diagram comparing induced and repressed genes between datasets. B) Log2 Fold Change comparison between different datasets. R2 correlation is indicated.

**Additional file 6: Fig. S5**. Translationally regulated members of mTOR, Wnt, NGF, and CREB pathways in developing neurons.

**Additional file 7: Figure S6**. Uncropped immunoblotting membranes for protein-of-interest and housekeeping antibodies. A) VAMP2. B) STMN3 and C) GPM6A.

**Additional file 8: Table S1**. Sequencing overview and gene expression data.

**Additional file 9:** **Table S2**. Gene Ontology analysis data.

**Additional file 10: Table S3**. Neuronal subcellular compartment data.

**Additional file 11: Table S4**. Translation efficiency of the translation-regulated genes found in our dataset in comparison with published data with cells in different stages of differentiation.

## Data Availability

All the sequencing data can be found at https://www.ncbi.nlm.nih.gov/sra, BioProject: PRJNA835330.
