# Supplementary material for: Genome-wide translation control analysis of developing human neurons
Source: Mol Brain. 2022 Jun 15;15:55. doi: 10.1186/s13041-022-00940-9 (PMC9199153; doi:10.1186/s13041-022-00940-9)
Supplement: Supplementary file 1 — Additional file 1: Supplemental experimental procedures. [file 13041_2022_940_MOESM1_ESM.pdf]

KEY RESOURCES TABLE

REAGENT or RESOURCE	SOURCE	IDENTIFIER
**Antibodies**		
Anti-TRA-1-60	Millipore Sigma	MAB4360
Anti-OCT4	Abcam	ab19857
Anti-Musashi 1	Abcam	ab154497
Anti-Nestin	Millipore Sigma	MAB5326
Anti-Synapsin I	Millipore Sigma	AB1543
Anti-MAP2	Abcam	ab5392
Anti-HOMER1	Synaptic systems	160003
Anti-GPM6A	Invitrogen	PA5-50350
Anti-STMN3	Invitrogen	PA5-53015
Anti-VAMP2	Invitrogen	MA5-32606
Anti-GAPDH	Cell Signaling	2118
**Cell lines**		
H9	WiCell	
HEK293T	ATCC	
**Stem cell/NPC culture**		
FGF2	Thermo Fisher Scientific	PHG0266
Dorsomorphin	Tocris	3093
Putrescine	Sigma-Aldrich	P5780-5G
Laminin	Thermo Fisher Scientific	23017015
Matrigel	Corning	354230
B-27™ Supplement (50X)	Thermo Fisher Scientific	17504044
N-2 Supplement (100X)	Thermo Fisher Scientific	17502048
SB 431542	Tocris	1614
Poly-L-ornithine	Sigma-Aldrich	P3655-50MG
Accutase	EMD Millipore	SCR005
DMEM/F-12	Thermo Fisher Scientific	31331093
Mtesr™1	STEMCELL Technologies	85850
**Purification kits**		
RNeasy	Qiagen	74104
miRNeasy	Qiagen	217004
Ribo-Zero	Illumina	MRZH11124
RNA Clean and concentrator	Zymo	R1015
**Reagents and Chemicals**		
Potassium Chloride	USB	20598
Paraformaldehyde	Sigma-Aldrich	P6148-500G
Titriplex EDTA	Merck - Millipore	108421
FORMAMIDE	Sigma-Aldrich	F9037-100ML
Dimethyl sulfoxide	Sigma-Aldrich	D2650-100ML
SUCROSE	Sigma-Aldrich	S0389-1KG
Urea	USB	75826
Magnesium chloride	USB	18641
Sodium Chloride	USB	21618
rATP, 10mM	Promega	P1132

DTT dithiothreitol	USB	15937
Tris base	Promega	H5135
Cycloheximide	Sigma-Aldrich	C7698-5G
Sybr Gold	Life technologies	S11494
Trizol	Ambion	15596018
Glycoblue	Invitrogen	AM9516
Bovine Serum Albumin	Sigma-Aldrich	A9418
**Enzymes/Recombinant proteins**		
SUPERase In	Thermo scientific	AM2696
Phusion	New England Biolabs (NEB)	M0530L
Poly(A) Polymerase	Thermo Scientific	AM2030
T4 PNK Kit	Thermo Scientific	ivgn230-4
SuperScript II	Invitrogen	18064-022
SuperScript IV	Invitrogen	18090010
TURBO DNase	Ambion (Life Tech)	AM2238
RNase I	Invitrogen	AM2295
RNase H	Affymetrix	70054Y
**Other**		
Dynabeads Oligo(dT)25	Invitrogen	61002
AGENCOURT AMPURE XP	Beckman Coulter	A63881
TRITC	Proteintech	SA00007-1
TRITC	Proteintech	SA00007-2
FITC	Proteintech	SA00003-2
ALEXA	Invitrogen	A22287
Prolong with DAPI	Life technologies	P36941
LuminoCt® SYBR® Green qPCR ReadyMix™	Sigma-Aldrich	L6544
Pierce™ BCA Protein Assay Kit	Thermo Scientific	23225
RIPA-lysis buffer	Thermo Scientific	89900
cOmplete™ Protease Inhibitor Cocktail	Roche	11697498001
SuperSignal™ West Femto Maximum Sensitivity Substrate	Thermo Scientific	34094
Spectra™ Multicolor Broad Range Protein Ladder	Thermo Scientific	26634

SUPPLEMENTAL EXPERIMENTAL PROCEDURES

Immunofluorescence

Cellular phenotype characterization of all cell types used in this work was done through immunostaining of classical markers such as TRA-1-60 (cell membrane) and OCT4 (nuclear) for hESC; Nestin (cytosolic) and Musashi1 (nuclear and cytosolic) for NPC; Map2 (cytosolic), Synapsin1 (pre-synaptic membrane) and Homer1 (post-synaptic membrane) for neurons. Shortly, cells were plated onto 13 mm glass coverslips pre-coated with Matrigel (hESC) or poly-L-ornithine/laminin (NPC and Neurons) in a 24-well plate. After 4 (hESC), 5 (NPC), and 30 (Neurons) days of culture, cells were fixed in 4%

paraformaldehyde for 20 minutes at room temperature. Next, cells were permeabilized with 0.1% Triton X-100 in 1X PBS for 15 minutes and then blocked with 2% BSA in 1X PBS for 4 hours at room temperature. The following primary antibodies, diluted in blocking solution, were added to the samples and incubated overnight at 4 °C: anti-TRA-1-60 (1:200), anti-OCT4 (1:200), anti-Nestin (1:200), anti-Musashi1 (1:1000), anti-Map2 (1:400), anti-Synapsin1 (1:400) and anti-Homer1 (1:200). The next day, cells were washed three times with 1X PBS and blocked again for 1 hour with 2% BSA in 1X PBS at room temperature. Species-specific secondary antibodies conjugated with TRITC-, FITC- or Alexa-dyes, also diluted in blocking solution, were incubated with samples for 1h at room temperature, protected from light. Cells were triple washed with 1X PBS and coverslips mounted using ProLong with DAPI.

RNA Extraction and Clean-up

RNA purification was done with Trizol reagent or by RNeasy, miRNeasy, or RNA Clean and Concentrator spinning columns using standard manufacturer protocols.

Sample processing for Ribosome Profiling

For cell lysis, media was removed from 10 cm plates and cells were scraped with 300 µl polysome lysis buffer (Tris-HCl 20 mM pH 7.5, KCl 1.5 mM, $MgCl_2$ 5 mM, 1% Triton X-100, DTT 1 mM) containing cycloheximide (CHX) 100 µg/ml. CHX was used in all buffers thereafter. Lysates were clarified by centrifugation for 10 minutes at 20.000 g. Lysate optical density was determined by Nanodrop at 260 nm absorbance and treated with 15U RNase I per OD for 30 minutes at 4ºC. This digestion protocol was previously published (Cenik et al., 2015) and preliminary compared by us with room temperature digestions and yields similar polysome profiles enriched 80S signal peak in sucrose gradients, reads lengths distribution (except for a 21 nt peak in RT digestions) and ribosome occupancy densities (data not shown). This protocol reduced ribosomal RNA contamination for ~70% to ~45% rRNA total reads in RT vs. 4ºC digestions (all also depleted with Ribo-zero), respectively. Digestion was stopped with SUPERase In and lysates were loaded in Type Ti-90 Beckmann rotor tubes containing 1 M sucrose cushion in polysome buffer. Ultracentrifugation was carried out for 5 hours, 55.000 RPM at 4ºC. The supernatant was discarded and the ribosomal pellet was resuspended with Qiazol. RNA was column purified and diluted in an equal volume of 2X formamide denaturing buffer (98% formamide, 10 mM EDTA, and 300 µg/ml bromophenol blue). The sample was denatured for 3 minutes at 80ºC and loaded in 15% Polyacrylamide TBE-Urea (8 M) gels. RNA was separated by gel electrophoresis for 2 hours at 200V. The gel was stained with SYBR Gold for 3 minutes and the footprints region (26-34 nt) was excised and recovered as described by Ingolia et al. (2012).

Sample processing for RNA sequencing

30 µg of Trizol purified RNA was digested with TURBO DNase for 10 minutes at 37ºC and RNA was column cleaned. 12 µl of Oligod(T) Dynabeads per sample were used for Poly(A) enrichment following manufacturer instructions. Enriched Poly(A) RNA was fragmented in Magnesium buffer (40 mM Tris-OAc, 100 mM KOAc, 30 mM $Mg(OAc)_2$) for 2’30” at 95ºC and cleaned by column purification.

NGS library construction

RNA templates (RNA fragments and footprints) were denatured for 90 seconds at 80ºC. 3' fragments were dephosphorylated with 10U T4 PNK for 60 minutes at 37°C following inactivation for 20 minutes at 65ºC. RNA fragments and footprints were polyadenylated with 2U Poly(A) RNA Polymerase in the dephosphorylation reaction tube (added with 250 mM NaCl and 1 mM rATP) for 30 minutes at 37°C. RNA was cleaned by column purification. Purified footprints were depleted of ribosomal RNA with Ribo-zero rRNA removal kit and cleaned by column.

Illumina adapters were inserted during the RT reaction using the Template-Switching technique as described (Hornstein et al., 2016) using Superscript II in ~20 µl reaction (1 µl/10 µM OligodT primer, 1 µl/10 µM template primer, 4 µl/5X First-Strand Buffer, 2 µl/0.1 M DTT, 1.2 µl/50 mM $MgCl_2$, 1 µL/dNTP Mix 10 mM and 1 µl 200U/µl SS II) for 60 minutes at 42°C and enzyme inactivated for 15 minutes at 75°C. cDNA was PCR amplified with Phusion High Fidelity for ~16 cycles using primers 5'-AATGATACGGCGACCACCGAGATCTACACTCTTTCCCTACACGACGCTCTTCCGATCT-3' (PE PCR) and 5'-CAAGCAGAAGACGGCATACGAGATNNNNNNGTGACTGGAGTTCAGACGTGTGCTCTTCCG-3' (Index, NNNNNN=barcodes). Libraries were size-selected and purified by polyacrylamide 10% TBE gel electrophoresis and AMPure beads. Library quality control and sequencing were done by LACTAD facility with Hiseq 2500 instrument, 100 bp SE run.

Polysome Analysis

Sucrose gradients were prepared with a Gradient Master 108, Biocomp. For HEK 293T cells, 15 cm plates at ~80% confluency were treated with CHX (100 µg/ml) for 30 minutes at 37ºC. Media was removed from plates, washed two times with 5 ml 1X PBS and cells were scraped with 250 µl polysome lysis buffer. NPCs (3 10cm plates) and Neurons (6 10cm plates) were treated with CHX for 10 minutes before harvest. Lysates were clarified by centrifugation at 20.000g for 10 minutes. Total RNA was purified from a small aliquot of the lysate with Trizol. The remaining of the samples was normalized (6OD A260U for HEK283T and 3OD A260U for NPC/Neuron) and loaded on top of the 10-50% sucrose gradients. Ultracentrifugation was carried at 36.000 RPM/4ºC for 3 hours, in an SW-41 rotor. Gradients were fractionated in a Brandel gradient fractionator system, meanwhile, the 254 nm absorbance was registered.

Sample processing for RT-qPCR

Total RNA (input) was purified with 1 ml Trizol, using standard manufacturer protocol. Sucrose gradient fractions were combined in 15 ml tubes in the following order: Free (fractions 1-5), the 80S (fractions 7-8), Low Polysomes (fractions 10-12) and Heavy Polysomes (fractions 14-16). Trizol was then added in a 1:1 ratio. After aqueous phase recovery, 2 volumes of 100% ethanol and 2 µl glycoblue were mixed in each pool for overnight precipitation at -20ºC. After centrifugation at 20.000 g for 30min at 4C, the pellets were resuspended in 50 µl water followed by the addition of 100 µl RNA Binding Buffer and purification on column with the kit RNA Clean & Concentrator kit (Qiagen). Total RNA and RNA obtained from sucrose fraction pools were reverse transcribed with SSIV using Oligod(T) in 20 µl final volume and then digested with 5U RNase H.

RT-qPCR

cDNA from the reverse transcription reaction was diluted in 1:50 in water. 4 μl of the diluted cDNA was used for RT-qPCR using forward and reverse primers (1 μM each) and LuminoCt® SYBR® Green qPCR ReadyMix for 40 cycles in CFX96 Touch Deep Well Real-Time PCR Detection System. Ct was determined by the CFX Maestro Software. Primers sequences for targets were: ATP6V0D1 (f:GAGGCCGTGAACATTGCTCAG r:GTTCATCTCGTCAAGGTCCTGC), NAPG (f:GGCAGTTGAATTACTAGGAAAAGCC r:TCTGAATAGAGAGTGCCGCCT), RAB3A (f:ACCATCACCACCGCATACTACC r:GGCCACGTTCTGATGACACC), ARG2 (f:GGTCCCGCTGCCATAAGAGAAG r:TAACCACCTCAGCCAGTTCCTG), GOT1 (f:GGACCTGGAACCACATCACTGA r: ACTGGATTTTGGTGACTGCTTCA), OAT (f:CCTTCTGATGTTGTAACTGCCGT r:GGACTCTCGAAGCTCATCCTCC), SRM (f:GCTGCACCTGGACCTCATCAA r:GGCTCCTGGAAGTTCGTGCT), CFL1 (f:GACTGCCGCTATGCCCTCTAT r:GTCAGCTTCTTCTTGATGGCGT) DPYSL2 (f:ACAGCGTTAAAACCATCTCTGCC r:GGACAATCTTCCCCTGGCTGAT), SS18L1 (f:CACAGTACCCCGGCTACCAG r:ATGTGTGTCCCTTACTGCTGGT) and for internal control: C11ORF58 (f:GTTCAGCCTCCCCAGACGAC r:TCCTGCACCCATAAGTCTCAAGA). C11ORF58 was selected as an internal control for the sucrose gradients since it has desired characteristics: a) it is highly expressed in NPC and Neurons; b) its translation efficiency is the same in NPC and Neurons; c) its transcription is the same between NPCs and Neurons. mRNA relative expression was calculated with the delta-delta Ct method by each fraction gene-of-interest and housekeeping expression normalization. 3 independent biological replicates were used for each cell type. Multiple unpaired, parametric t-test was performed for Total, Free, 80S, Low Poly and High Poly fractions followed by Benjamini-Hoechberg correction. Statistical significance was set at alpha<0.05.

Immunoblotting

Cells from 10 cm plates were washed two times with ice-cold 1X PBS, then lysed with 300 μl RIPA buffer supplemented with 1X protease inhibitor (Roche). Lysates were cleared by centrifugation at 20.000 g for 10 min and protein concentration was determined by the Pierce™ BCA Protein Assay Kit. SDS-polyacrylamide gels were loaded with 20 μg (GPM6A and STMN3) or 50 μg (VAMP2) protein which were separated by electrophoresis at 50V for 4 hours. Proteins were transferred to PVDF membranes for 25 minutes at 10V using Bjerrum Schafer-Nielsen buffer in Trans-Blot® SD Semi-Dry Transfer Cell. Membranes were blocked for 1 hour with 5% BSA TBST then incubated overnight at 4ºC with primary antibodies in 5% BSA TBST with the following dilutions: anti-VAMP2 (1:500), anti-GPM6A (1:1000), and anti-STMN3 (1:1000). Secondary HRP incubation was done in 1:10000 dilution for 1 hour at room temperature. SuperSignal™ West Femto Maximum Sensitivity Substrate was used for chemiluminescence and signal was detected with ChemiDoc Imaging Systems. After stripping, membranes were incubated with the anti-GAPDH (1:5000) antibody. Densitometry of bands was performed using GelQuant.NET software. 4 independent biological replicates were used per condition.

Bioinformatics and NGS data analysis

Clean reads. Initially, the quality of the raw reads was assessed using the software FastQC version 0.11.5 [1]. Initially, the software Cutadapt version 2.4 was used to trim any remaining Illumina adapter that wasn't removed during the demultiplex with the following parameters '-a GATCGGAAGAGCACACGTCTGAACTCCAGTCACNNNNNNATCTCGTATGCC GTCTTCTGCTTG --trim-n --minimum-length 9'. Because the protocol used to build the libraries add poly-T sequences at the beginning of the reads and poly-C at the end, an in-house script was developed to remove these bases. To trim bases with poor quality the software Trimmomatic version 0.36 was used with the parameters 'TRAILING:3 SLIDINGWINDOW:4:15 MINLEN:9'. One of the characteristics of Riboseq libraries is the contamination, mainly with rRNA, and sometimes with tRNA. To remove the contaminating reads it was built a reference with all subunits of human rRNA and tRNA from nuclear and mitochondrial genomes. The reads were aligned against the contamination reference using the software Bowtie2 version 5.4.0 with the parameters '--local -L 15 -i S,1,0.5 -k 1'. Only the reads that do not align to the contaminant reference were maintained to downstream analysis.

Expression Analysis. To assess the gene expression, it was built a reference with all the human transcripts (Figure S2C) but removing pseudogenes and the 5' UTR and 3' UTR of the coding transcripts. The software Kallisto version 0.44.0 was used to build the index with the parameter '--kmer-size=21' and to quantify the expression with the parameters '--bias -b 30 --single --rf-stranded -l 30 -s 10'. The statistical analysis was conducted with the R package DESeq2 version 3.7. for RNA sequencing, Ribosome Profiling, and TE data. A threshold of FC>2 and FDR<0.01 was defined as the statistical significance. An additional filter that weighed the 3 independent FDR calculations (RNAseq, Riboseq, and TE) was employed (Figure 2B and S2C) to fit an obligatory alpha threshold in all measures for the TE data set. This filter guarantees that Translational Efficiency changes are necessarily followed by Riboseq or RNAseq differential expression changes defined by DEseq2.

GO and compartment data analysis

A list of genes classified as regulated was tested for enrichment compared with backgrounds using standard DAVID (functional chart annotation), IPA (canonical pathways), and SynGO parameters. Data successfully retrieved from human axonal transcriptome, monosome/polysome preferentially translated transcripts and the synaptic bouton proteome were tested for enrichment using Fisher's exact test followed by Bonferroni's p-value correction.

SUPPLEMENTARY FIGURES AND TABLES

Figure S1. hESC differentiation into NPCs, and neuronal differentiation. A) NPC generation steps, as detailed in methods. B) Light microscopy images showing the time-course phenotype of neural cells after NPC differentiation induction by FGF removal.

Figure S2. RNA sequencing expression heatmap and clustering classification of canonical progenitor, neuroepithelial, differentiation, neuronal, synaptic, and glutamatergic markers.

Figure S3. Quality control of the NGS libraries. A) Optimization of RNase I digestion protocol for ribosome footprint generation with Hek293T samples. B) PCA of NGS biological replicates produced in this study. C) Flowchart of filters applied for Translational Efficiency classification. D) IGV aligned STAR reads tracks comparing Riboseq and RNAseq libraries.

Figure S4. Comparison of transcriptionally regulated genes between our dataset and previously published data, obtained with cells in different days of NPC differentiation into neurons. A) Venn diagram comparing induced and repressed genes between datasets. B) Log2 Fold Change comparison between different datasets. R2 correlation is indicated.

Figure S5. Translationally regulated members of mTOR, Wnt, NGF, and CREB pathways in developing neurons.

Figure S6. Uncropped immunoblotting membranes for protein-of-interest and housekeeping antibodies. A) VAMP2. B) STMN3 and C) GPM6A.

Table S1. Sequencing overview and gene expression data.

Table S2. Gene Ontology analysis data.

Table S3. Neuronal subcellular compartment data.

Table S4. Translation efficiency of the translation-regulated genes found in our dataset in comparison with published data with cells in different stages of differentiation.

REFERENCES

Andrews, S. (2015). FASTQC A Quality Control tool for High Throughput Sequence Data. Babraham Inst.

Bray, N.L., Pimentel, H., Melsted, P., and Pachter, L. (2016). Near-optimal probabilistic RNA-seq quantification. Nat. Biotechnol.

Bolger, A.M., Lohse, M., and Usadel, B. (2014). Trimmomatic: A flexible trimmer for Illumina sequence data. Bioinformatics.

Cenik, C., Cenik, E.S., Byeon, G.W., Grubert, F., Candille, S.I., Spacek, D., Alsallakh, B., Tilgner, H., Araya, C.L., Tang, H., et al. (2015). Integrative analysis of RNA, translation, and protein levels reveals distinct regulatory variation across humans. Genome Res.

Hornstein, N., Torres, D., Das Sharma, S., Tang, G., Canoll, P., and Sims, P.A. (2016). Ligation-free ribosome profiling of cell type-specific translation in the brain. Genome Biol.

Ingolia, N.T., Brar, G.A., Rouskin, S., McGeachy, A.M., and Weissman, J.S. (2012). The ribosome profiling strategy for monitoring translation in vivo by deep sequencing of ribosome-protected mRNA fragments. Nat. Protoc.

Langmead, B., and Salzberg, S.L. (2012). Fast gapped-read alignment with Bowtie 2. Nat. Methods.

Love, M.I., Huber, W., and Anders, S. (2014). Moderated estimation of fold change and dispersion for RNA-seq data with DESeq2. Genome Biol.
Marchetto, M.C.N., Carromeu, C., Acab, A., Yu, D., Yeo, G.W., Mu, Y., Chen, G., Gage, F.H., and Muotri, A.R. (2010). A model for neural development and treatment of rett syndrome using human induced pluripotent stem cells. Cell.
Martin, M. (2011). Cutadapt removes adapter sequences from high-throughput sequencing reads. EMBnet.Journal.
