# Supplementary material for: Genome-wide translation control analysis of developing human neurons
Source: Mol Brain. 2022 Jun 15;15:55. doi: 10.1186/s13041-022-00940-9 (PMC9199153; doi:10.1186/s13041-022-00940-9)
Supplement: Supplementary file 2 — Additional file 2: Fig. S1. hESC differentiation into NPCs, and neuronal differentiation. A) NPC generation steps, as detailed in methods. B) Light microscopy images showing the timecourse phenotype of neural cells after NPC differentiation induction by FGF removal. [file 13041_2022_940_MOESM2_ESM.pdf]

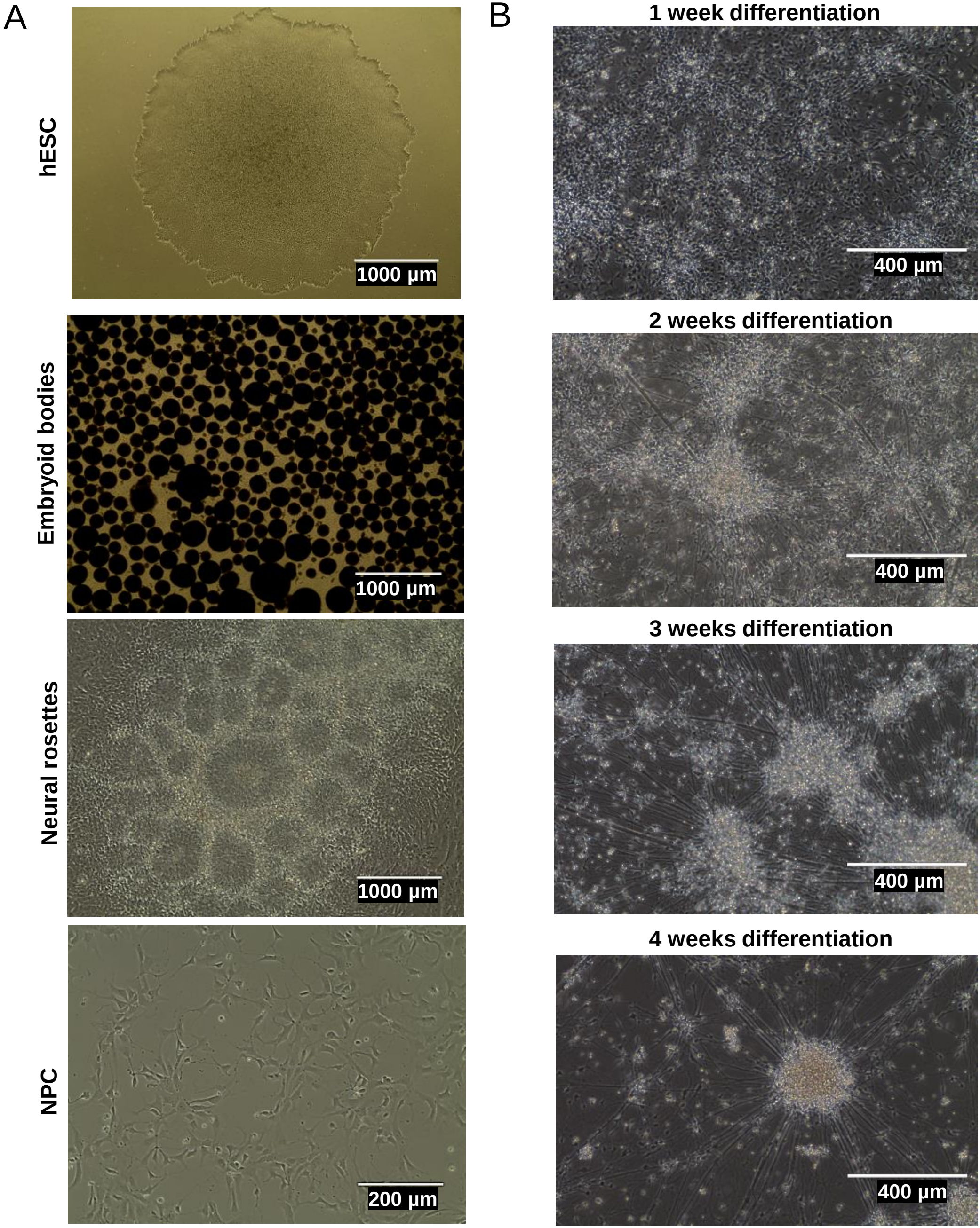
A
hESC
1000 µm
Embryoid bodies
1000 µm
Neural rosettes
1000 µm
NPC
200 µm
B
1 week differentiation
400 µm
2 weeks differentiation
400 µm
3 weeks differentiation
400 µm
4 weeks differentiation
400 µm
