# Supplementary figures and images for: Genome-wide translation control analysis of developing human neurons
Source: Mol Brain. 2022 Jun 15;15:55. doi: 10.1186/s13041-022-00940-9 (PMC9199153; doi:10.1186/s13041-022-00940-9)

A

RNA-seq

-1 0 1

Row Z-Score

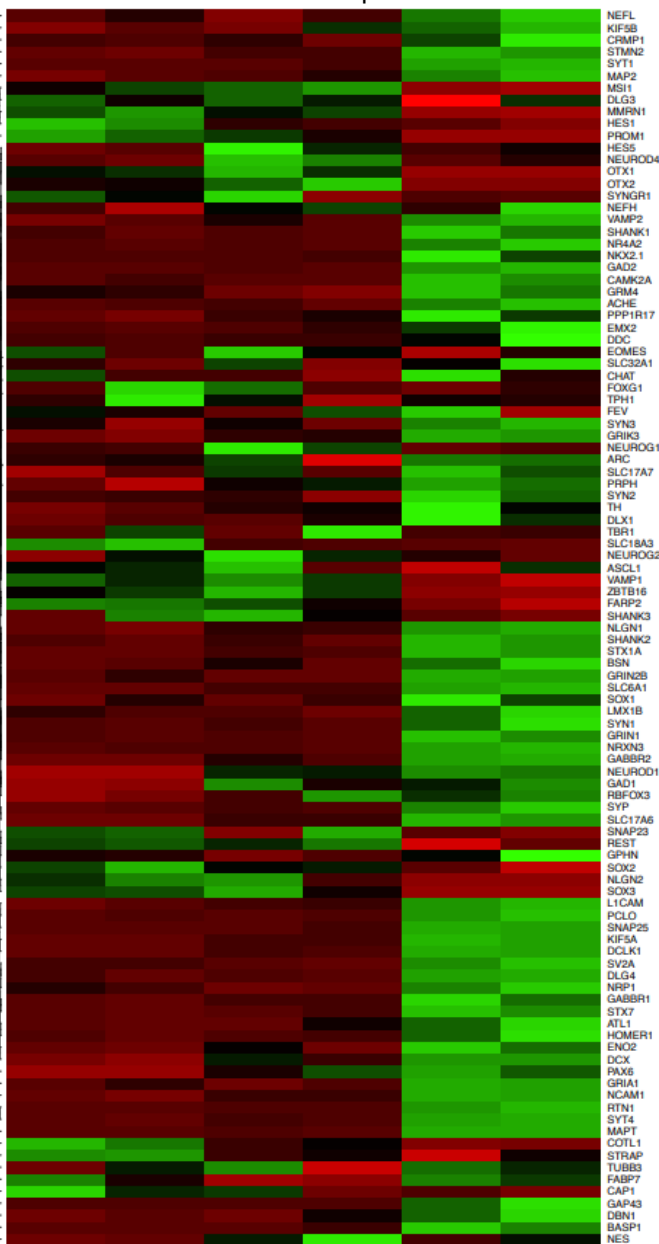

Supplement: Supplementary file 3 — Additional file 3: Fig. S2. RNA sequencing expression heatmap and clustering classification of canonical progenitor, neuroepithelial, differentiation, neuronal, synaptic, and glutamatergic markers. [file 13041_2022_940_MOESM3_ESM.pdf]

A

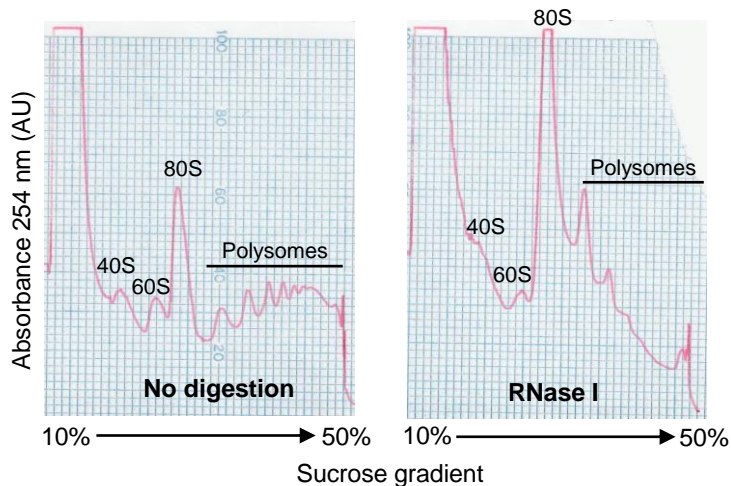

B

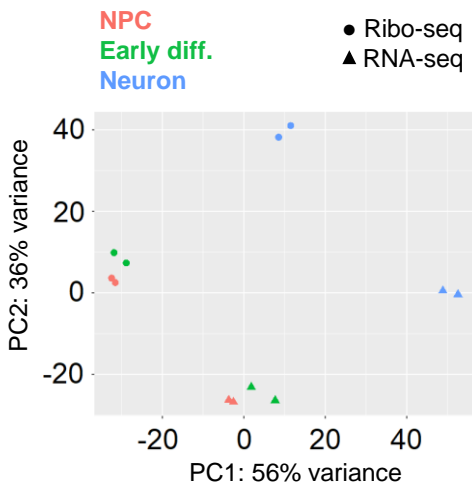

C

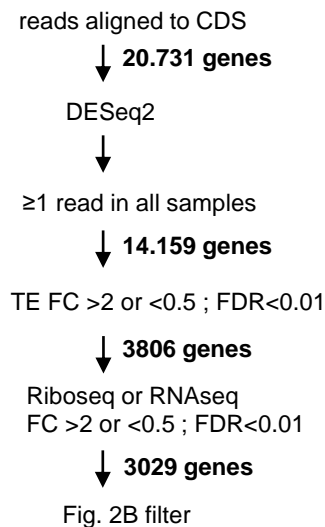

D

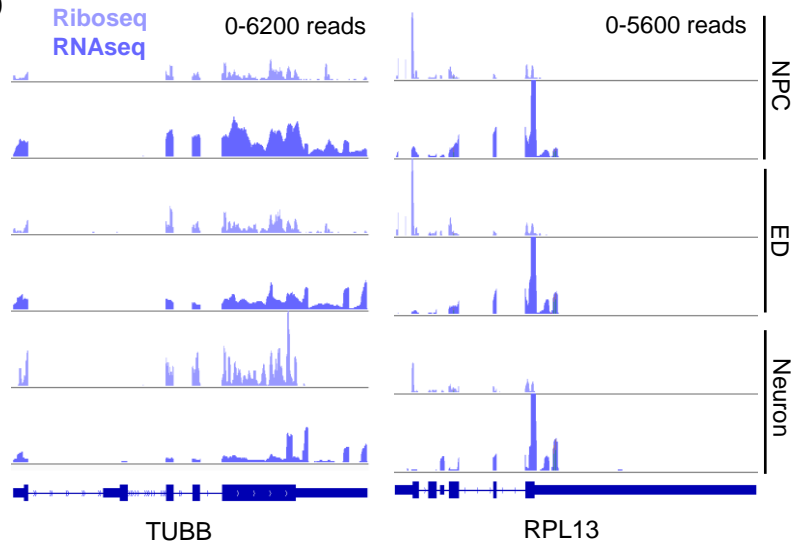

Supplement: Supplementary file 4 — Additional file 4: Fig. S3. Quality control of the NGS libraries. A) Optimization of RNase I digestion protocol for ribosome footprint generation with Hek293T samples. B) PCA of NGS biological replicates produced in this study. C) Flowchart of filters applied for Translational Efficiency classification. D) IGV aligned STAR reads tracks comparing Riboseq and RNAseq libraries. [file 13041_2022_940_MOESM4_ESM.pdf]

A)

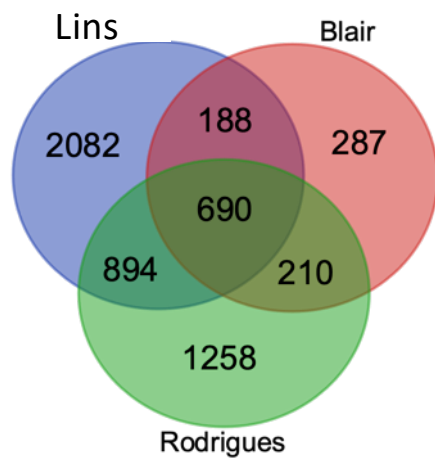

Transcriptionally induced genes

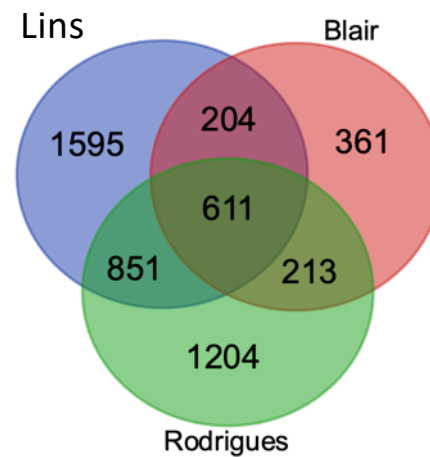

Transcriptionally repressed genes

B)

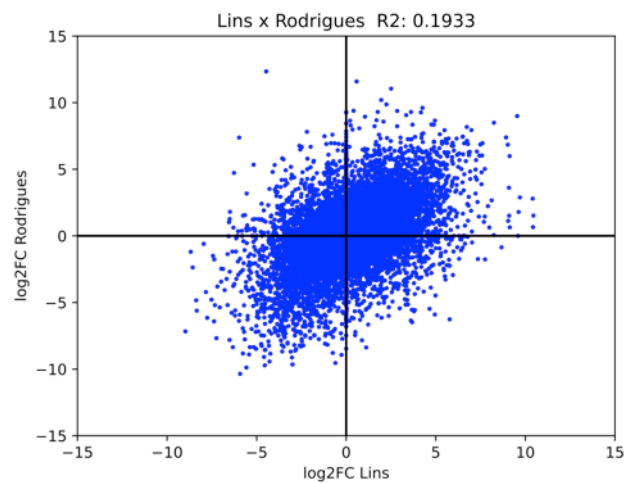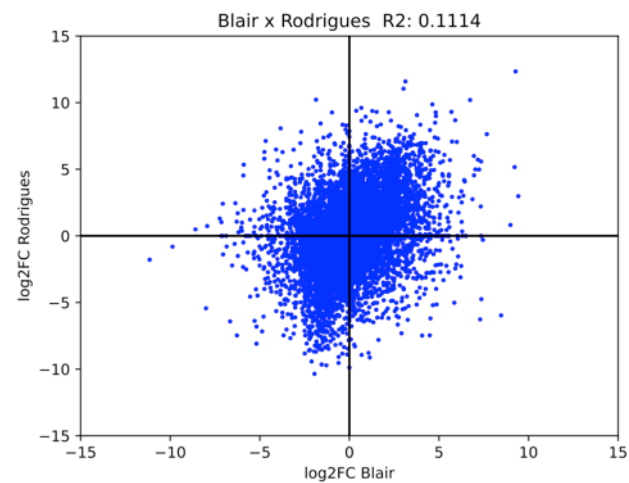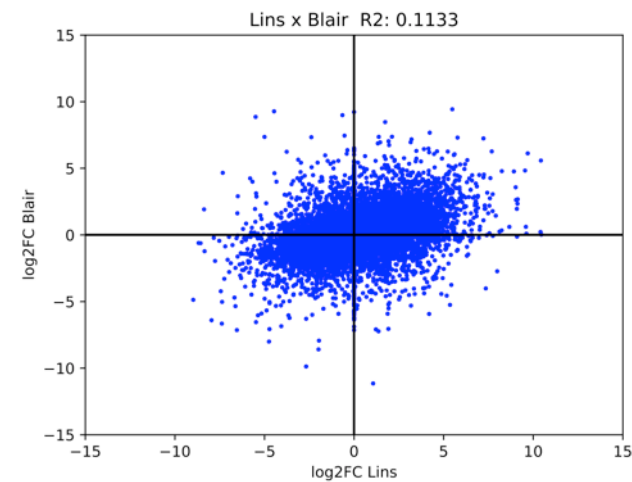

Supplement: Supplementary file 5 — Additional file 5: Fig. S4. Comparison of transcriptionally regulated genes between our dataset and previously published data, obtained with cells in different days of NPC differentiation into neurons. A) Venn diagram comparing induced and repressed genes between datasets. B) Log2 Fold Change comparison between different datasets. R2 correlation is indicated. [file 13041_2022_940_MOESM5_ESM.pdf]

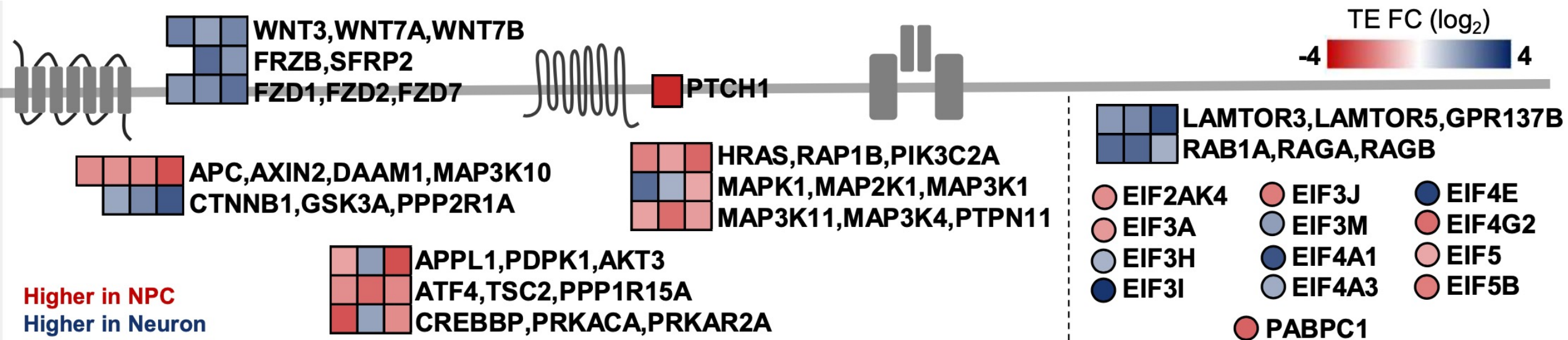

Supplement: Supplementary file 6 — Additional file 6: Fig. S5. Translationally regulated members of mTOR, Wnt, NGF, and CREB pathways in developing neurons. [file 13041_2022_940_MOESM6_ESM.pdf]

A)

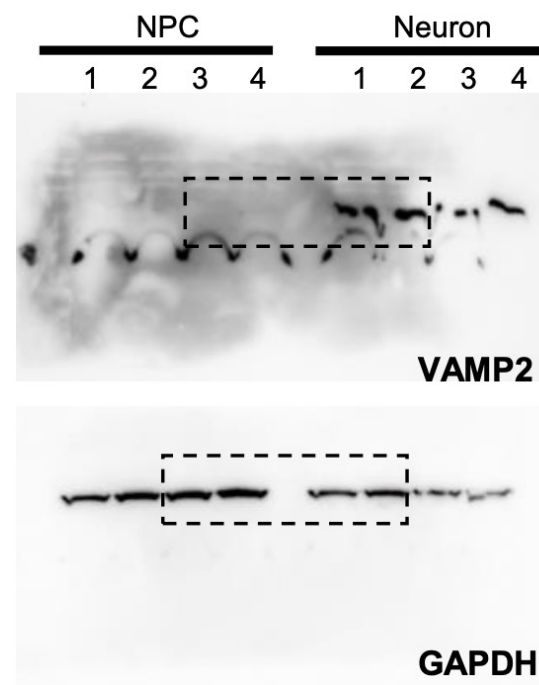

Figure 3G

B)

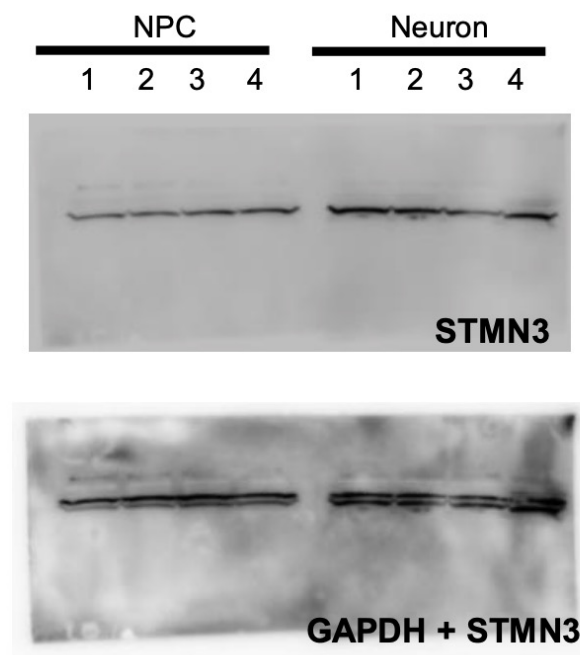

Figure 5E

C)

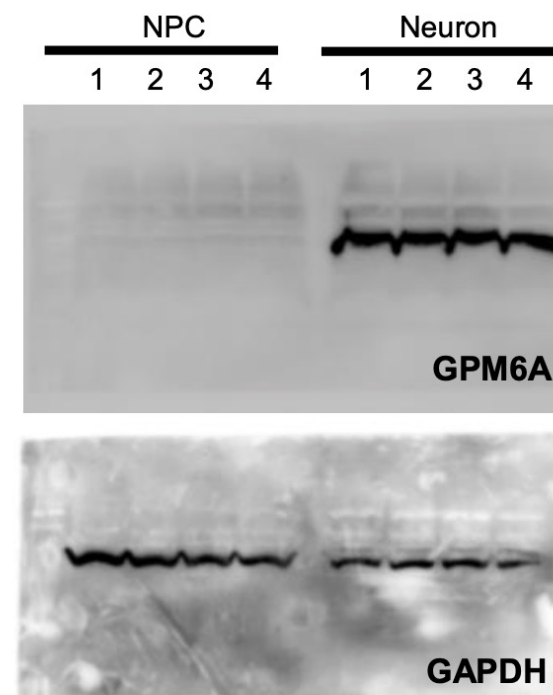

Figure 5E

Supplement: Supplementary file 7 — Additional file 7: Figure S6. Uncropped immunoblotting membranes for protein-of-interest and housekeeping antibodies. A) VAMP2. B) STMN3 and C) GPM6A. [file 13041_2022_940_MOESM7_ESM.pdf]
